# Eye Movements during Dynamic Scene Viewing are Affected by Visual Attention Skills and Events of the Scene: Evidence from First-Person Shooter Gameplay Videos

**DOI:** 10.16910/jemr.14.2.3

**Published:** 2021-10-21

**Authors:** Suvi K. Holm, Tuomo Häikiö, Konstantin Olli, Johanna K. Kaakinen

**Affiliations:** University of Turku, Turku, Finland

**Keywords:** eye movement, eye tracking, individual differences, dynamic scene, events, attention, video game, eSports, gameplay video

## Abstract

The role of individual differences during dynamic scene viewing was explored. Participants (N=38) watched a gameplay video of a first-person shooter (FPS) videogame while their eye movements were recorded. In addition, the participants’ skills in three visual attention tasks (attentional blink, visual search, and multiple object tracking) were assessed. The results showed that individual differences in visual attention tasks were associated with eye movement patterns observed during viewing of the gameplay video. The differences were noted in four eye movement measures: number of fixations, fixation durations, saccade amplitudes and fixation distances from the center of the screen. The individual differences showed during specific events of the video as well as during the video as a whole. The results highlight that an unedited, fast-paced and cluttered dynamic scene can bring about individual differences in dynamic scene viewing.

## Introduction

The spectating of E-sports, i.e. competitive digital game contests, is becoming immensely popular (10; 26). Many E-sports games consist of first-person shooter (FPS) games, in which players move around rapidly. Whilst avoiding enemy fire, the players must shoot at correct targets that stay put for only milliseconds. There seem to be individual differences in how much of a challenge following this type of a visual scene presents: for some people, just watching it is enough to bring about motion sickness (64), while E-sports stars and active players seem to follow the game effortlessly. These types of games can therefore offer an excellent chance to explore how certain visual attention skills affect the viewing of a cluttered, dynamic scene.

In this paper, we will focus on individual differences in certain visual attention skills that have been known to be affected by action games such as FPS games. However, our focus is not on whether these skills are affected by gaming, but rather how differences in these skills manifest during watching of gameplay videos. We are particularly interested in how different individuals react to specific game events, such as aiming at a target or getting hit by the enemy fire. As we examine these reactions during viewing of a 6-minute game video, we will also be able to describe some general eye movement patterns associated with gameplay spectating, for example, how viewing patterns change across time.

### Individual Differences in Visual Attention Skills

Recent research has shown that people who are trained with particularly cluttered dynamic scenes, namely videogames, tend to have better visual attention skills (e.g. 7). Multiple studies have shown that active videogame players have improved target detection, i.e., they are more likely to attend to visual targets that others might miss, and they also detect targets from a wider area in the scene (22; 25; 72). Even though these studies have sometimes been criticized (see 30; 55), they suggest that there is a link between individual differences in visual attention skills and performance in cognitively demanding dynamic scenes. For example, Bavelier and Green (6) posit that games such as first-person shooters encourage attentional shifting, updating and inhibition. In short, participants need to move from focused to divided attention, update main goals and sub-goals, as well as ignore non-targets. Particularly relevant to eye movement patterns, Bavelier and Green (6) make the case that players need to move from a diffused attentional state, such as monitoring for enemies in the periphery of the screen, to a focused attentional state, such as when engaging with enemies. What remains an open question is whether individuals who have good visual attention skills in the first place (that is, without having rehearsed videogame playing) are better at dealing with the fast-paced cluttered visual environment of video games, which is why they might get more attracted to them. In the present study we aim to answer this question by examining whether some of the cognitive skills identified to be enhanced as a product of videogame playing are involved also during passive watching of videogaming videos, and whether the individual differences in these skills affect eye movements.

Previous research shows that there are individual differences in eye movements that seem to be consistent across different visual tasks (2; 4; 12; 29; 51; 56). Hayes and Henderson (27) showed that individual differences in general intelligence, speed of processing and working memory capacity affect scan paths during viewing of static natural scenes. For example, higher cognitive ability as indicated by various measures was associated with a tendency to focus attention more centrally within the scene.

In the present study, we examined whether individual differences in three skills noted to be enhanced through action video game playing are reflected in eye movements during free viewing of unedited videogame videos. These skills are multiple object tracking, visual search, and susceptibility to attentional blink. Next, we discuss these skills and why we think they are relevant for the particular first-person shooter game we used in this study.

### Multiple Object Tracking

Multiple Object Tracking (MOT) (49) refers to a skill of tracking many visually identical-looking objects simultaneously while they move. The ability to do this is especially important in dynamic scene viewing (40). For example, it may be a key element in following the unfolding events during watching a videogame video of a war scene with multiple moving enemy soldiers, typical for a FPS game. Oksama and Hyönä (45) found that there are individual differences in MOT performance, and that these individual differences correlate with other high-order cognitive skills, namely temporary spatial memory and attention switching.

Research on eye movements during the MOT task shows that when fixations land to a central location between tracked targets instead of directly on top of targets, MOT gets easier (20, 21; 78). This strategy may be based on viewers trying to keep track of a group as a whole instead of scanning individual targets serially (20, 21). In the context of viewing gameplay videos, this might be reflected in eye movements as fewer, longer and more centrally located fixations, as well as shorter saccade amplitudes as individuals with good MOT skills might be able to track the moving objects in the scene without scanning all possible fixation targets serially, that is, switching from target to target.

### Visual Search

Visual search refers to the ability to quickly find a target object among distractors (18; 74). Finding target objects is harder if the scene is cluttered (53). As a general rule, the more cluttered the visual search array is, the more fixations tend to get longer and saccades shorter during the task (52).

There are individual differences in eye movement patterns in visual search (9). Namely, viewers tend to adopt one of two strategies when they search for a target among distractors: a covert search strategy characterized by few eye movements and utilization of peripheral vision, and an overt strategy in which the screen is scanned with many eye movements (9). The overt strategy tends to lead to worse performance in visual search (8, 9), leading to Boot et al. (8) aptly calling the phenomenon “the more you look, the less you see”. All in all, these two styles of visual search tend to be fairly stable preferences among the viewers who use them (9).

We consider visual search abilities to be important for watching FPS game videos because finding targets, namely enemies, among distractors, such as team mates, is relevant for following the game events. Most FPS gaming videos contain considerable clutter and murkiness of the environment, making visual search all the more difficult. When it comes to eye movements, participants who are faster at VS might adopt the covert strategy, which may show as fewer but more centrally located fixations, and possibly longer fixation durations and shorter saccade amplitudes, as participant do less scanning of the visual environment.

### Attentional Blink

Attentional blink (AB) refers to a phenomenon in which participants find it hard to see a target if a non-target is shown right before the actual target (17). The magnitude of the AB effect varies between individuals (38), and some also seem to be unaffected by the phenomenon (39). The role of detecting targets rapidly is especially important when one considers fast-paced videos, such as videogaming videos. Videogames, especially FPS games, are notorious for being extremely fast-paced. If the viewer is not able to detect important targets (such as enemies) that are presented only rapidly one after each other or is unable to distinguish between targets and distractors (such as team mates), following the video becomes difficult. In eye movements, less susceptibility to the phenomenon, that is, better ability to detect serially rapidly appearing targets, might show as more fixations with shorter durations and less central locations, as well as longer saccade amplitudes.

Individual differences in MOT and VS abilities and susceptibility to AB are likely to manifest in reaction to game events in the cluttered and fast-paced gaming video: for example, when the protagonist in the video is aiming at a target or gets hit by enemy fire, or when there is a sudden change of scenery. In addition to individual differences in reactions to different game events, there could also be general scanning tendencies that develop across time, as viewers get used to the gaming scene. Before discussing how different game events might be reflected in viewers' eye movements, we will discuss relevant previous research on visual attention during dynamic scene viewing.

### Visual Attention During Dynamic Scene Viewing

Studying visual attention when perceiving dynamic scenes has so far received less attention from eye movement research than the study of static stimuli (52; 66). This may have to do with the complexity of dynamic scenes, which may contain many visual properties, spatial relationships and events including movement and actions (36). However, certain phenomena related to eye-movements have been noted related to dynamic scenes.

People make eye movements to direct the high acuity foveal vision to locations that need particular scrutiny to provide information they cannot process through the parafovea or the peripheral vision (28; 52; 66). Previous research on static scene viewing suggests that there are two different "modes" of scene viewing: ambient (global) and focal (local) (67). The ambient mode is characterized by short fixations and long saccades as people scan around the image. In the focal mode, the fixations get longer and the saccades get shorter, i.e. the gaze tends to stay put more and the area being scanned gets smaller (47; 68). During static image viewing, the pattern of fixations usually goes from ambient to focal mode, as has been reported in various studies (3; 11; 23; 33). There is some evidence that the ambient to focal processing phenomenon may present to some extent during viewing of dynamic scenes as well, although it has so far been noted to be relative to scene cuts, scene onsets and events rather than to the entire duration of a dynamic scene (19; 46; 57). We will explore this more when we discuss the role of game events on visual attention during gameplay viewing in the next section. For now, we will focus on general eye movement phenomena related to dynamic scene viewing.

It has been established that participants tend to keep their eyes on the center of the screen more during watching of dynamic in comparison to still scenes (16; 61). This may be due to the “blink and you will miss it” style of fast-paced video material: participants watching videos do not have as much time to focus on specifics as participants watching still images. As the gist of a scene can be understood quickly (28; 52), and as sometimes parafoveal or peripheral vision is enough to determine what an object is (48), viewers can create basic understanding of what is going on in the scene even if the progression of the video material is fast-paced and there is no time to do further scanning. Moreover, if the eyes are fixated on the center of the screen, it allows equal chance for detecting a point of interest from the periphery of vision to which to make a saccade next. In this sense, the center of the screen seems to form a base from which saccades leave from and where they come back to after a target needing further inspection in the periphery of the screen has been scanned.

Besides this tendency for center bias, the gazes of viewers watching dynamic scenes tend to be far more clustered to specific objects of the scene, indicating attentional synchrony, that is, less individual differences in gaze locations (42; 58). Some of the most important low-level features known to grab attention are object color, motion, orientation and size (73). During dynamic scene viewing, when a scene contains both stationary as well as moving objects, moving objects tend to grab attention (15; 73). The role of motion as a salient feature that captures attention has also been noted in studies featuring films and edited video clips (14; 35; 42). Likewise, during free viewing of unedited videos of natural scenes, people tend to gaze more at areas that flicker (61). Moreover, sudden onsets or appearances of new objects tend to capture attention (e.g. 75). Motion and change are often informative for the viewer, and informative parts of a dynamic scene tend to be fixated more than parts that are homogenous and stable (70).

Another feature that captures attention in scenes is people (61), which shows as participants looking less at visually salient areas when the scene contains humans than when it does not contain humans (54). As humans often also move around in films, they effectively grasp attention (60).

Moreover, emotional content of a dynamic scene modulates eye movements, leading to fixations landing on a narrower area during emotion-inducing scenes than during non-emotional scenes (65). Furthermore, if video content is perceived as negative, low-level saliency is not as important in guiding fixations (54).

In summary, previous research shows that during dynamic scene viewing fixations tend to be clustered around the center of the screen (16; 61). Eye movements of the viewers are synchronized to focus on movement (14; 15; 35; 42; 73), and especially on human motion (60). Moreover, emotional scene content narrows the dispersion of the eye movements across the scene (65). One special characteristic of longer dynamic scenes such as movies and gaming videos is that they often contain events and transitions between those events. Next, we will discuss the role of these events in guiding attention during gameplay viewing.

### The Role of Game Events on Visual Attention during Gameplay Viewing

Many videogames may have a limited but recurring content of specific events. A small body of studies has annotated what they considered key events for FPS videogames. For example, Lang et al. (34) identified a “Hunting phase vs. killing phase”, i.e. whether the protagonist was looking for enemies or was in active battle with them (see also 71). Nacke et al. (43) considered the player firing a gun, the player getting hurt and the player dying to be the most meaningful events in an FPS game. In a similar vein, Ravaja et al. (50) indicated the most important events to be wounding the opponent, killing the opponent, the player’s character being wounded and the player getting killed. Lopes et al. (37) considered a player entering a room to be a meaningful event boundary. What is characteristic of these events is that they constitute of visually salient features, such as human motion or distinct color (red for blood) appearing on the screen, which are likely to capture viewers' attention. However, understanding what these visually salient features mean in the context of the gameplay requires at least rudimentary comprehension of the narrative of the game.

Humans tend to divide a continuous stream of actions into events that have a beginning and an end, and these events are key components of perception, attention, and memory (77). This segmentation happens automatically and is a continuously ongoing component of perception (77). For example, the “coarse grain” event of taking over a building in a FPS game video might consist of several “fine grain” events, such as scanning the environment for enemies and eliminating them, advancing towards the entrance of the building while avoiding enemy fire, and entering the building via a corridor. Viewers of the video are likely to create event models out of these types of actions, and these models integrate features derived from different sensory modalities and determine where attention is guided next and which features are inhibited (e.g., 59). The event models are also affected by previous event schemata, that is, learned statistical or important knowledge about events such as which patterns of activity are likely to follow and what kind of goals the actors might have (77). Zacks et al. (77) suggest that viewers constantly make predictions about future input in the dynamic scene on the basis of event models and that is what drives attention to certain parts of the scene.

Previous evidence shows that event models impact eye movements during dynamic scene viewing (19; 57). Smith et al. (57) showed that saccade frequencies decreased before fine grain event boundaries and increased after the boundary passed during perception of unedited natural dynamic scenes. Pannasch (46) presented results indicating that abrupt scene cuts such as displaying a new environment in a new scene or extending the environment of the current scene via horizontal camera movements brings about the ambient mode of scanning, characterized by longer saccades and shorter fixations. In a study on viewing of unedited videos of actors performing various tasks, the beginning of an event (e.g., changing a tire to a car) was associated with an ambient mode of viewing (19). As the event progressed, the participants’ eye movements became more focal, characterized by longer fixations and shorter saccades (19).

In summary, events and event boundaries play an important role in guiding eye movements during viewing of dynamic scenes, such as game videos. Typical game events are characterized by visually salient features, and in combination with the relevance of these events in the narrative of the gameplay, they can be expected to trigger either ambient or more focal modes of viewing. In the present study, we examined individual differences in how viewers react to different types of game events.

### Aims of the Current Study

The aim of our study was to investigate whether visual attention skills identified to be enhanced as a product of FPS videogame playing are reflected in eye movements during passive viewing of gameplay videos, and specifically, in eye movement responses to specific gameplay events. In order to examine how visual attention skills, and not gaming experience, is associated with viewing patterns of gaming videos, we recruited participants who had very little prior experience of gaming.

As FPS games are typically fast-paced and present a cognitively demanding visual environment (6), they are optimal for examining individual differences in viewing behavior. We utilized gameplay videos recorded of one of the most popular FPS games, PlayStation 3 version of Call of Duty: Modern Warfare 2 (Activision, 2009). As viewing of E-sports and gameplay videos is getting increasingly popular (10; 26), gameplay videos provide an ecologically valid context for examining individual differences in dynamic scene viewing.

Even though eye movements are already implemented in videogame design as controls for games (1; 69), there are hardly any academic studies on eye movements during videogame viewing, especially independent of playing, and the present study should be considered as exploratory in nature. We expected that individual differences in visual attention tasks that have been linked with FPS gaming performance (6) are reflected in how viewers react to gameplay events on the video. We also expected that there may be individual differences in how viewers' eye movement patterns develop across time, as they adjust to the cluttered and fast-paced visual environment of the gameplay video.

## Methods

### Participants

Participants were recruited via an internet survey on preferred game dynamics and time spent playing videogames. The survey was posted to several student organizations’ mailing lists around the city of Turku, Finland. 199 respondents answered the survey. We set out to recruit participants with as little gaming experience as possible. Two questions of the survey focused on this in particular: 1. “Think about the past year. How many hours did you spend playing videogames on a typical week? Try to estimate your weekly playing time even if you did not play every week”, and 2. “According to your estimation, how much have you played videogames during your whole gaming history?” For question 1, participants gave their answer in hours: “During a typical week, I played videogames for XX hours.” For question 2, a 5-point Likert scale was utilized (1 = not at all, 5 = a lot).

We invited those respondents who had left their contact information to the experiment (N = 124). Forty participants eventually took part in the laboratory experiment. Two of the participants’ datasets had to be discarded because of calibration issues, leading to a final dataset of 38 participants (11 men, 27 women, M_age_ = 28.29 years, SD_age_ = 7.14 years). The participants played on average 1.28 hours per week (SD = 2.38, range = 12) and they estimated they had accumulated fairly little gaming experience during their gaming history (M = 2.5, SD = .98).

### Apparatus

Eye movements were recorded using EyeLink 1000+ (SR Research Ltd., Ontario, Canada) with a 500 Hz sampling frequency. A remote mode with a target sticker placed on a participant’s forehead to track head movements was utilized. The camera recorded x and y coordinates of the participants’ dominant eye’s movements. All tasks were presented using a 24” Benq XL2420Z screen with 144 Hz refresh rate. The participants sat at a distance of 70 cm from the screen and the camera was positioned right in front of the screen.

### Materials

#### Gameplay Videos

We recorded gameplay videos from the PlayStation 3 version of Call of Duty: Modern Warfare 2 (Activision, 2009), a popular FPS game. The single player campaign mode of the game was used. The campaign mode is composed of missions (levels) in which the protagonist has to follow the leader of the troop and act according to their commands.

For this experiment, we created a gameplay video of four missions by recording a gamer playing through the game missions “Wolverines”, “Exodus”, “Gulag”, and “Whiskey Hotel”. All videos were 6 minutes long and taken from the beginning of the mission without the intros. The player did not die in any of the videos. The videos had a resolution of 1920x1080 and a frame rate of 30.

#### Selection of Gameplay Events

The videos were broken down into specific events. First, four independent coders scored the ”Gulag” gameplay video for events that appeared on the video frame by frame. The coders followed Eisenberg and Zacks’ (19) classification of coding “a meaningful action”. The coders made notes of each individual event they considered meaningful to a playing or watching experience. During the process, it became evident that there were repetitive events that could be classified and described, such as when the protagonist got hit. Because of this repetitive nature and the visually differing qualities of the events, we decided to code the events into categories.

After coding the first video, coding of the event categories was based on consensus between the independent raters. The initial event classification consisted of the following events: (1) advancing, (2) turning, (3) start of firing (enemy or own team, but not self), (4) aiming at a target, (5) reloading weapon, (6) changing weapon, (7) protagonist gets hit (blood on visor), (8) unexpected salient events (helicopters, tanks, explosions, etc.), (9) change of environment, (10) picking and throwing a grenade, (11) using a laser to mark targets, (12) someone else uses a laser, and (13) taking cover.

After this selection, timestamps of the events were documented for all four of the missions by noting down at which frame the events started. This was done by two independent scorers. Both timestamped the events for all four videos individually and then checked together that all relevant events were included. The ‘advancing’ category was separated to cases in which (1) the protagonist moves and (2) the team members move and the protagonist stays stationary.

After this initial classification, we followed Järvelä et al.’s (31) guidelines of using videogame events as stimuli, i.e. that they should be properly isolated from other events and appear frequently. It was determined that 2 seconds would be a sufficient time to detect changes in eye-movements and create enough isolation between different events. Thus, we only considered events that were separated from the next coded event by 2 seconds. Events that happened before two seconds had passed since the previous coded event were therefore discarded from the analyses. We then counted frequencies of the remaining events and ended up discarding any event categories that did not happen at least 8 times in any of the videos. This process eliminated 7 of the event classes.

**Figure 1.**
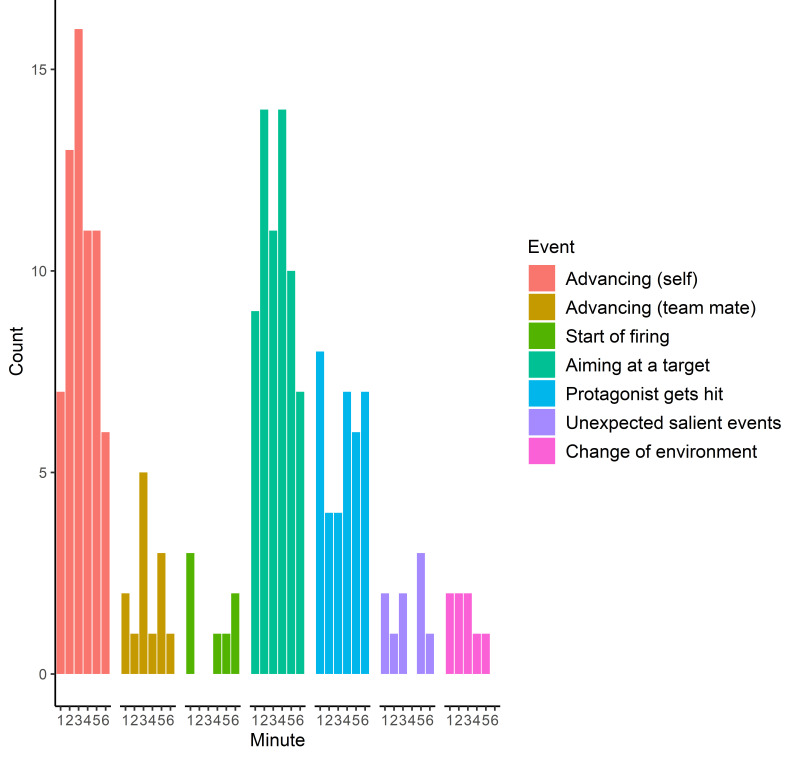
Counts of different events (summed across all videos) as a function of time (6 minutes).

The final event classes consisted of: (1) Advancing (self), (2) Advancing (team mate), (3), Start of firing, (4) Aiming at a target, (5) Protagonist gets hit (bloodied visor), (6) Unexpected salient events, and (7) Change of environment. The frequencies of these events in each video are presented in Table 1. The frequencies of these events during all videos across time (6 minutes, the length of the videos) are illustrated in Figure 1.

**Table 1.** Frequencies of events in different game missions.

	Mission			
Event	W	E	G	WH
Advancing (self)	42	27	20	31
Advancing (team mate)	10	10	13	10
Start of firing	8	4	7	6
Aiming at a target	25	47	26	49
Protagonist gets hit (bloodied visor)	6	15	10	23
Unexpected salient events	47	28	9	30
Change of environment	7	4	2	10

Note. Missions abbreviated as follows: W = Wolverines, E = Exodus, G = Gulag, WH = Whiskey Hotel.

#### Descriptions of the Gameplay Events

Advancing (self) is an event in which the protagonist is moving forward towards a new area and is not currently engaged in active battle. This event is often presented as running forward and scanning for enemies by turning of the protagonist’s head.

Advancing (team mate) is an event in which the protagonist is fairly stationary but their team mate(s) start(s) to move towards a new area. This event is often presented as team mates running past the protagonist when there is a pause in active battle. Notably, if both the protagonist itself and the teammates were moving forwards together, the event was coded as ‘Advancing (self)’.

Start of firing is an event in which either a team mate or an enemy opens fire after a calm period, therefore starting a new phase of battle. It is noteworthy that this event type refers only to either team mates or enemies opening fire, not the protagonist itself. If it was the protagonist that started the firing, it was coded as ‘Aiming at a target’.

Aiming at a target is an event in which the player aims down the sight of their weapon at an enemy. As the protagonist aims at a target, the character looks down the gun’s sight, creating a distinctive visual scenario in which a crosshair appears at the center of the screen and the view zooms in on the target. Aiming at a target is almost always followed by shooting, which in turn leads to either wounding or, in most cases, killing of the enemy. Because the time frame between aiming and shooting is very short, we combined these events under ‘Aiming at a target’.

Protagonist gets hit (bloodied visor) is an event in which the protagonist gets wounded. This is shown as a flinch response of the protagonist, blurry vision and various amounts of blood filling the screen, depending on how badly the protagonist gets hit.

Unexpected salient events refer to situations in which curious objects that may capture attention appear suddenly. These objects included, for example, airplanes, tanks, or parachutists. Moreover, visually salient objects such as blinking screens or big explosions were coded under this event type.

Change of environment consists of events in which the environment changes considerably – for example, the protagonist enters a tunnel or a house.

### Cognitive Tasks

#### Attentional Blink Task (AB)

In the AB task utilized for this experiment, participants were instructed to look for an L-shaped target stimulus presented on a screen. In addition to the target stimulus, there were 3 distractor stimuli that looked alike to the target but were positioned at different angles. In each trial of the task, the participant looked at a focus point in the middle of the screen. Meanwhile, two out of four possible stimuli were flashed quickly in rapid succession to each other. A mask covered the stimuli rapidly after they were flashed. The participant had to indicate whether the actual target had been present in the trial or not. There were altogether 96 trials. The number of correct answers was recorded. The AB task utilized in this experiment is available online at PsyToolkit.org (62, 63). Notably, the original experiment contains 104 trials, of which 8 trials were discarded from the current experiment. During these eight trials, two stimuli are presented at the same time. The experiment was re-scripted for E-Prime 2.0 (Psychology Software Tools) using the open script files at PsyToolkit’s website (www.psytoolkit.org, 2019) and presented using E-Prime 2.0. Internal consistency of the AB task was investigated using a split-half method. We obtained a Spearman-Brown coefficient of .77

#### Visual Search Task (VS)

In the VS task used in this experiment, the participants had to find a red letter ‘T’ amongst other ‘T’-shaped letter distractors that were either the same color as the target but upside down, or a different color (blue) than the target. The participants were instructed to respond as quickly as possible by pressing a key on the keyboard when they had seen the target. They were not to respond at all if the target was not present in the trial. There were 48 trials containing either 5, 10, 15 or 20 items to search through. Half of the trials (24) contained a target. The number of correct answers as well as the reaction time of the correct answers was recorded. In our analyses, we used the reaction time of the correct answers of the trials in which the target was present. The VS task utilized in this experiment is available online at PsyToolkit.org (62, 63). The experiment was re-scripted for E-Prime 2.0 (Psychology Software Tools) using the open script files at PsyToolkit’s website (www.psytoolkit.org, 2019) and presented using E-Prime 2.0. Internal consistency of the AB task was investigated using a split-half method. We obtained a Spearman-Brown coefficient of .64.

#### Multiple Object Tracking Task (MOT)

In the MOT task used in this experiment, the participants needed to keep track of five moving pictures while not tracking identical-looking pictures. Each trial started with the screen showing 10 identical pictures of a face. After this, 5 of the faces blinked to indicate that they were to be tracked. This was followed by the blinking stopping and all 10 faces moving around fast and in random directions for 6 seconds. After the 6 seconds had passed, the faces stopped moving and the participant had to click those faces they thought they were tracking. There were 15 trials in the task and the number of fully correct answers (all 5 target faces identified) was recorded. The MOT task was scripted and presented using E-Prime 2.0. Internal consistency of the MOT task was investigated using a split-half method. We obtained a Spearman-Brown coefficient of .46.

### Procedure

The participants signed an informed consent form and received instructions for the experiment at the start of the experiment. They filled out a number of questionnaires (the results of which will not be reported here), after which electrodes were attached to their face and feet for psychophysiological recordings (the results will not be reported here). Then, the participants completed the cognitive tasks. After having finished the tasks, the participants moved on to play one mission of the game and watch one gameplay video of the same game. Only the video watching part will be reported here. The conditions of watching and playing were counterbalanced: every other participant started by playing and every other by watching a video. The mission of the video and the playing condition were not the same for the same person, i.e. each participant watched a video of a certain mission of the game and played another mission. The missions in question were pre-determined by using a latin square method to ensure that the frequencies of the mission presentations were as even as possible. Eye movements were only recorded during the video watching condition. At the end of the experiment, participants filled out surveys about how familiar they were with the game on a Likert scale ranging from 1 (not at all familiar) to 5 (very familiar). The mean rating for familiarity was 1.63 (SD = 1.02, two participants had missing values), indicating the participants were not familiar with the game. The participants also filled out surveys about how difficult playing the game was and what their emotional state was like during playing and watching, but these results will not be reported here. The duration of the whole experiment was around 2 hours.

### Statistical Analyses

The statistical analyses focused on individual differences in visual attention skills and how those differences affected the dependent variables, namely number of fixations, fixation durations, fixation distances from the screen center, and saccade amplitudes during watching of gameplay videos. Furthermore, specific events during the videos were included in the analyses.

Before building our models, we explored correlations between the visual attention tasks. MOT and AB correlated very weakly (*r_s_* = .07, *p* = .67, *N* = 40), MOT and VS correlated weakly (*r_s_* = -.21, *p* = .20, *N* = 40), and VS and AB correlated weakly (*r*(38) = -.35 *p* = .03). Because there were no strong correlations between the tasks, we decided to include all of the visual attention tasks in the models.

The data were analyzed with linear mixed models using the lme4 package (version 1.1.23, 5) in the R program (version 3.6.1, R Core Team, 2019). For the number of fixations, we generated a generalized linear mixed model utilizing the Poisson distribution. For each dependent variable (eye movement measure), we carried out an analysis in which the fixed effects were time from the start of the video, cognitive tasks (AB, VS, MOT), game events, the interactions between the different cognitive skills and time, and the interactions between different cognitive skills and game events. Time and the scores of the cognitive tasks were centered: the mean was 0 and the unit was SD. For the events, the baseline was the “other” category (time points that fell outside the coded events). Participants and the particular videos (game missions) they watched were included in the models as random effects. Figures were drawn using the interactions (version 1.1.3), effects (version 4.1.4) and ggplot2 (version 3.3.0) packages in R.

We used four eye movement measures: number of fixations per each minute, fixation duration, fixation distance from screen center and saccade amplitude. The number of fixations were summed across each minute of the video. The rest of the eye movement measures were analyzed at the level of individual fixations/saccades. Fixation duration is simply the duration of individual fixations in ms. Fixation distance from screen center refers to the distance in pixels from the center point of the screen. Saccade amplitude refers to the length of the saccade in angular degrees.

Before analyzing the fixation measures, we first removed all fixations that did not fit inside the screen’s coordinates (0 < x < 1920, 0 < y < 1080). Then, all fixations that deviated in duration for more than 3 SD from each participant’s personal mean were removed before the analyses. The amount of outliers removed was 2.03%. After removing outliers, a logarithmic transformation was carried out for fixation duration.

For the saccade amplitude analyses, all saccades that did not start or end inside the screen’s coordinates (0 < x < 1920, 0 < y < 1080) were removed. Outliers that deviated for more than 3 SD from each participant’s personal mean were also removed, resulting in the removal of .43% of saccades. After this removal, a logarithmic transformation was carried out for saccade amplitudes.

## Results

### Descriptive Statistics for Cognitive Tasks

Descriptive statistics of the visual attention tasks are presented in Table 2. For the AB task and the MOT tasks, we used the percentage of correct answers out of all answers in the analyses. For the VS task, we used reaction time for correct answers.

**Table 2.** Descriptive statistics of visual attention tasks.

Skill	Mean	SD	Min	Max
AB correct answers (%)	68.90	18.15	30.00	96.00
VS correct answers RT (ms)	1002.05	181.47	716.46	1550.64
MOT correct answers (%)	67.33	13.15	40.00	100.00

### Number of Fixations

The model for the number of fixations is reported in Table 1 in Appendix 1. Only statistically significant results will be discussed here. There were main effects of Time and Event on number of fixations. The number of fixations decreased as the video progressed. Moreover, the number of fixations was lower during the events of ‘Advancing (self)’, ‘Advancing (team mate)’, ‘Aiming at a target’, and ‘Protagonist gets hit (bloodied visor)’ than during the baseline. Instead, during the events of ‘Start of firing’ and ‘Unexpected salient events’, the number of fixations was higher than during the baseline.

**Figure 2.**
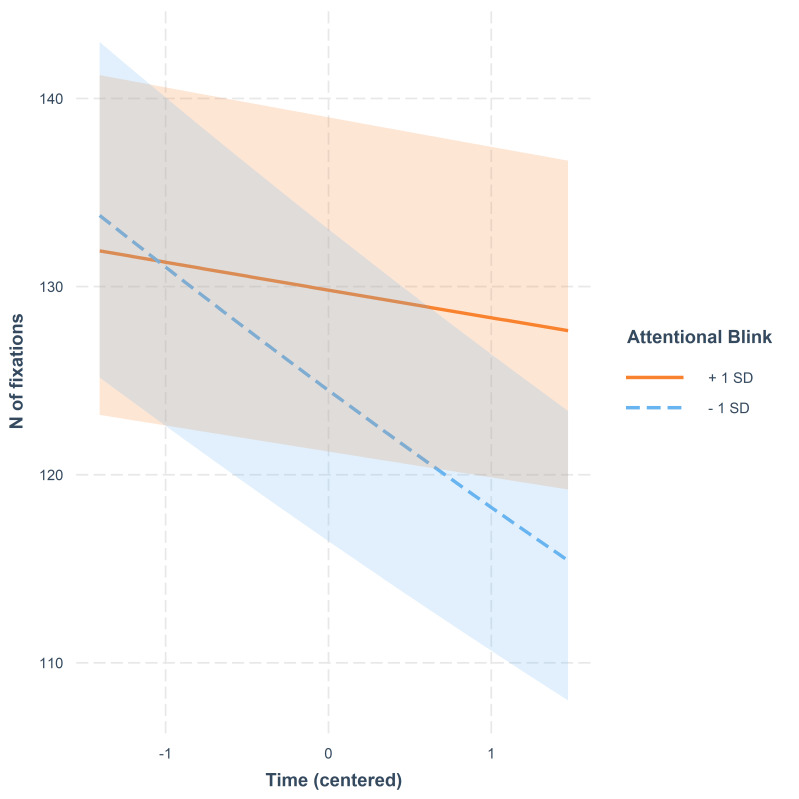
Number of fixations as a function of time. The lines represent model estimates at values one standard deviation below and one standard deviation above the mean in AB. The shaded areas denote 95% confidence intervals.

**Figure 3.**
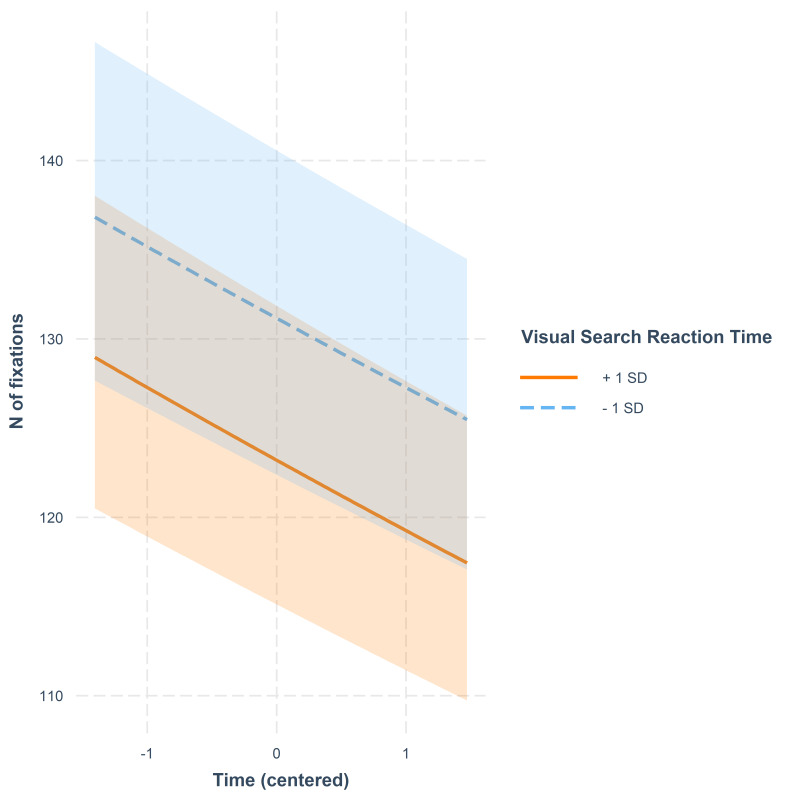
Number of fixations as a function of time. The lines represent model estimates at values one standard deviation below and one standard deviation above the mean in VS reaction time. The shaded areas denote 95% confidence intervals.

**Figure 4.**
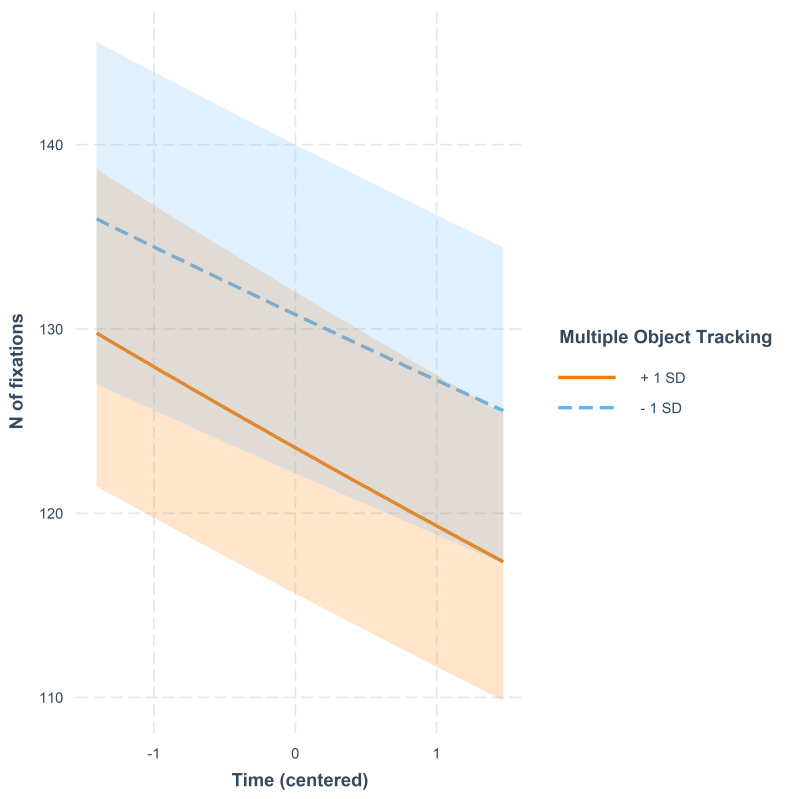
Number of fixations as a function of time. The lines represent model estimates at values one standard deviation below and one standard deviation above the mean in MOT. The shaded areas denote 95% confidence intervals.

There were interactions between AB * Time, VS * Time, and MOT * Time. A high score in AB was connected with the number of fixations staying relatively stable over time; instead, a low score indicated a steep decrease in number of fixations as the video progressed. This trend is illustrated in Figure 2. The weak interaction between VS and Time is presented in Figure 3. A slow reaction time in VS was connected with a slightly steeper decrease in number of fixations over time when compared to those with a fast reaction time. There was a weak interaction between MOT and Time: a high score in MOT was connected with a slightly steeper decrease in number of fixations over time when compared to a low score in MOT. This trend is illustrated in Figure 4.

**Figure 5.**
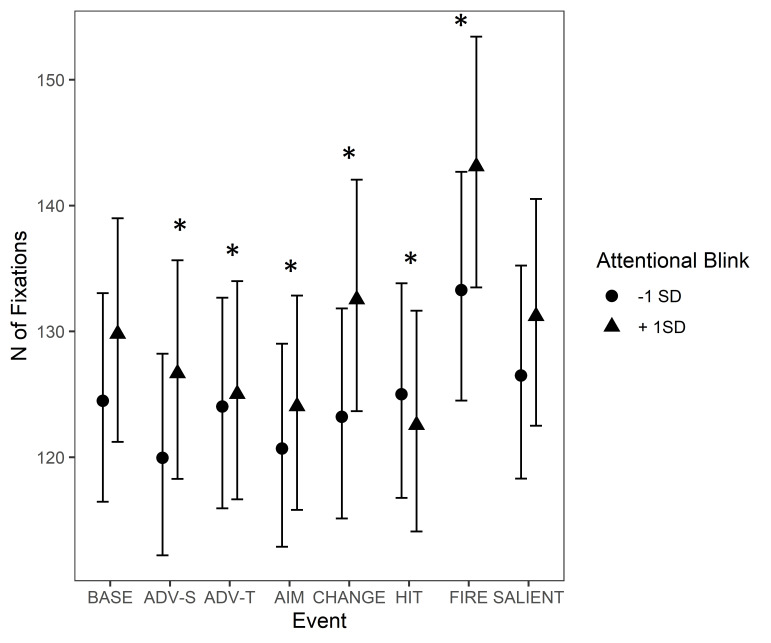
Number of fixations during game events. The circle and triangle symbols represent model estimates at values one standard deviation below and one standard deviation above the mean in AB. The error bars denote 95% confidence intervals. Note. Significant interactions between Attentional Blink and Event Type are marked with an asterisk (*). The events are abbreviated as follows: BASE = Baseline, ADV-S = Advancing (self), ADV-T = Advancing (team mate), AIM = Aiming at a target, CHANGE = Change of environment, HIT = Protagonist gets hit (bloodied visor), FIRE = Start of firing, SALIENT = Unexpected salient events.

All the cognitive tasks had interaction effects with some of the game events. The Event type * AB interaction is illustrated in Figure 5. AB had an interaction with all events except for ‘Unexpected salient events’. In the baseline condition, participants with a higher AB score made more fixations than those with a lower AB score, even though this effect was not significant. When compared to the baseline, the effect of AB on the Event type increased during ‘Advancing (self)’, ‘Change of environment’, and ‘Start of firing’, indicating that during these events, the participants’ skills in AB affected the number of fixations more than during baseline. Namely, the higher the score was in AB, the more fixations participants tended to make during these events. Instead, the effect of AB decreased during ‘Advancing (team mate)’, ‘Aiming at a target’, and ‘Protagonist gets hit (bloodied visor)’ when compared to the baseline, indicating that skills in AB had less impact for number of fixations during these events than during baseline.

**Figure 6.**
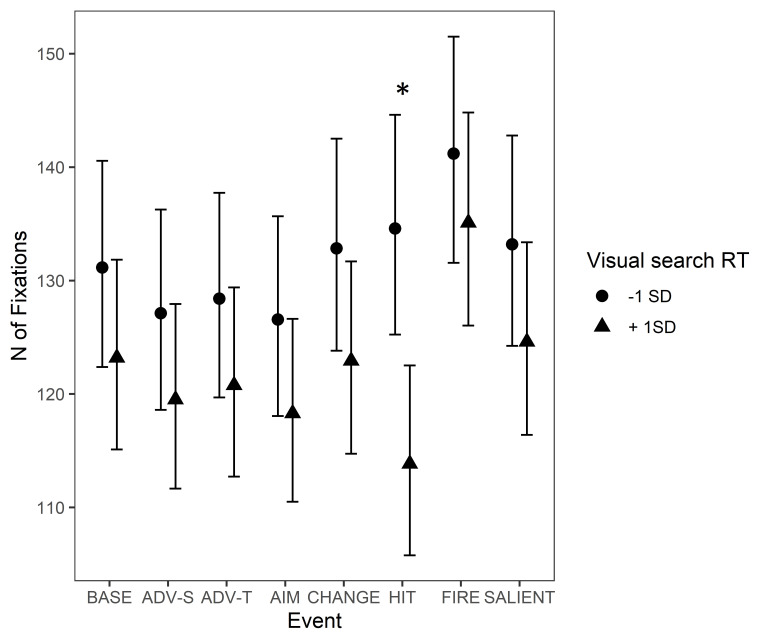
Number of fixations during game events. The circle and triangle symbols represent model estimates at values one standard deviation below and one standard deviation above the mean in VS reaction time. The error bars denote 95% confidence intervals. Note. Significant interactions between Visual Search and Event Type are marked with an asterisk (*). The events are abbreviated as follows: BASE = Baseline, ADV-S = Advancing (self), ADV-T = Advancing (team mate), AIM = Aiming at a target, CHANGE = Change of environment, HIT = Protagonist gets hit (bloodied visor), FIRE = Start of firing, SALIENT = Unexpected salient events.

There was an interaction between VS and ’Protagonist gets hit (bloodied visor)’ but not the other ame events. During ‘Protagonist gets hit (bloodied visor) the effect of VS increased compared to the baseline: the slower the reaction time in VS, the less fixations participants tended to make during this event, whereas participants with a faster reaction time made a somewhat similar amount or slightly more fixations than during the baseline. This effect is illustrated in Figure 6.

**Figure 7.**
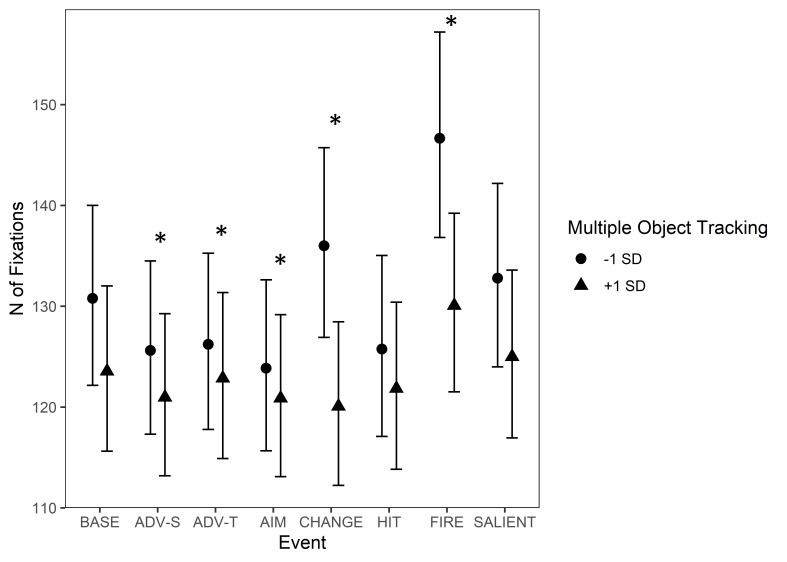
Number of fixations during game events. The circle and triangle symbols represent model estimates at values one standard deviation below and one standard deviation above the mean in MOT. The error bars denote 95% confidence intervals. Note. Significant interactions between Multiple Object Tracking and Event Type are marked with an asterisk (*). The events are abbreviated as follows: BASE = Baseline, ADV-S = Advancing (self), ADV-T = Advancing (team mate), AIM = Aiming at a target, CHANGE = Change of environment, HIT = Protagonist gets hit (bloodied visor), FIRE = Start of firing, SALIENT = Unexpected salient events.

There were interactions between MOT and all other events except for ‘Character gets hit (bloodied visor)’ and ‘Unexpected salient events’. In the baseline condition, participants with a higher MOT score made fewer fixations than those with a lower MOT score, even though this effect was not significant. When compared to the baseline, the effect of MOT on the Event type increased during ‘Start of firing’ and ‘Change of environment’, indicating that during these events, the skill differences in MOT had a bigger effect on number of fixations than during baseline. Namely, the lower the score was in MOT, the more fixations there tended to be during these events. Instead, the effect of MOT decreased during the events of ‘Advancing (self)’, ‘Advancing (team mate)’ and ‘Aiming at a target’ when compared to the baseline. The effects for MOT and Events are illustrated in Figure 7.

### Fixation Duration

The results of the model are reported in Table 2 in Appendix 1. Neither time nor the three cognitive skills had main effects on fixation duration. However, there were main effects for the individual events. During the ‘Advancing (team mate)’ and the ‘Unexpected salient’ events, participants made shorter fixations than during baseline. Instead, during the ‘Aiming at a target’ event, participants made longer fixations than during baseline.

**Figure 8.**
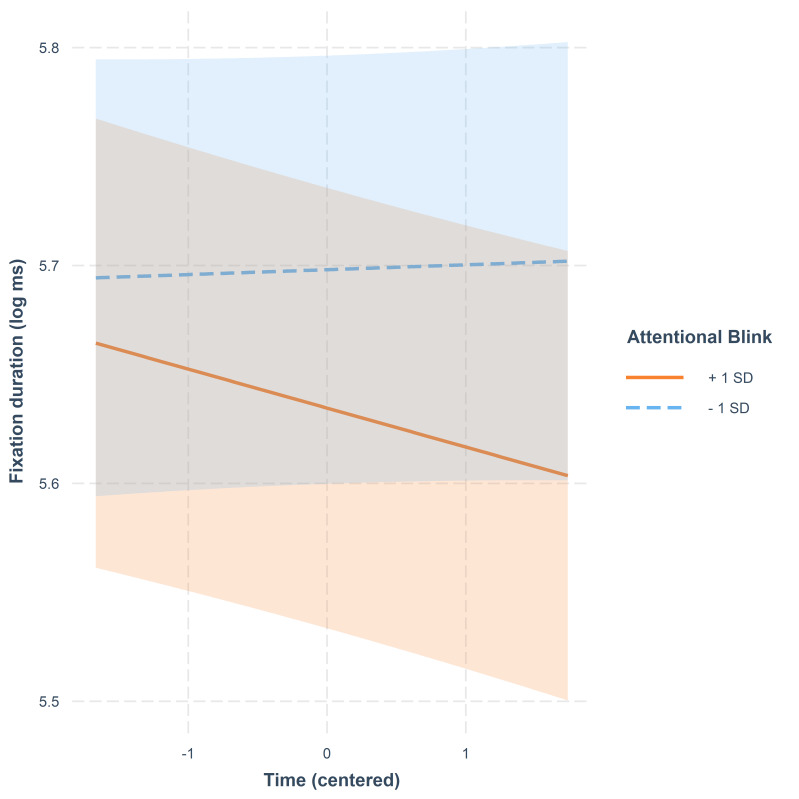
Fixation duration (log-transformed ms) as a function of time. The lines represent model estimates at values one standard deviation below and one standard deviation above the mean in AB. The shaded areas denote 95% confidence intervals.

There was an interaction between AB and Time. This interaction is illustrated in Figure 8. For those participants who scored high on the AB task, their fixation durations showed a decreasing trend towards the end of the video. Instead, the participants with a low score showed a stable or slightly rising tendency in fixation duration towards the end of the video.

**Figure 9.**
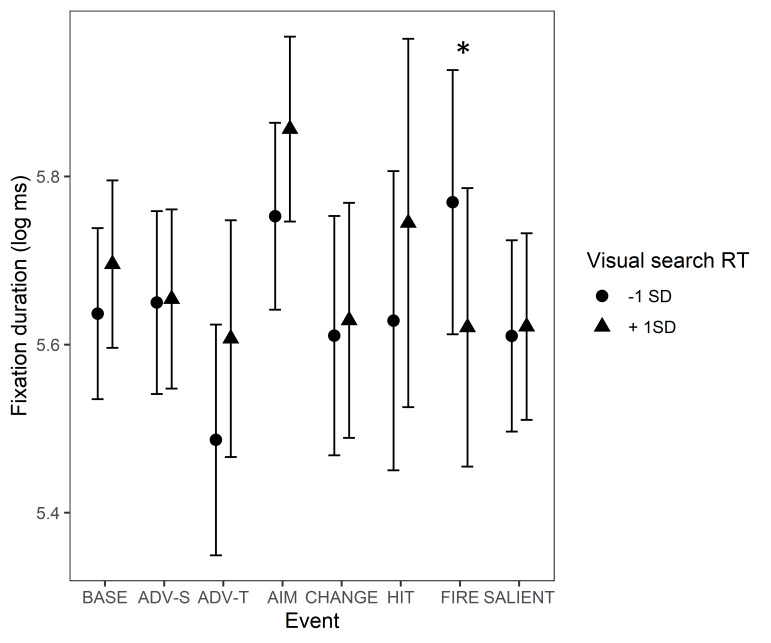
Fixation durations during game events. The circle and triangle symbols represent model estimates at values one standard deviation below and one standard deviation above the mean in VS. The error bars denote 95% confidence intervals. Note. Significant interactions between Visual Search and Event Type are marked with an asterisk (*). The events are abbreviated as follows: BASE = Baseline, ADV-S = Advancing (self), ADV-T = Advancing (team mate), AIM = Aiming at a target, CHANGE = Change of environment, HIT = Protagonist gets hit (bloodied visor), FIRE = Start of firing, SALIENT = Unexpected salient events.

Some of the cognitive skills also had interaction effects with the game events. VS had an interaction with the ‘Start of firing’ event. This effect is illustrated in Figure 9. In the baseline condition, slower reaction times in VS were associated with a trend of longer fixation durations. However, during the ‘Start of firing’ event, the effect of VS reversed, indicating that the slower a participant was in VS, the shorter their fixation durations were during ‘Start of firing’. Instead, the faster a participant was in VS, the longer the fixations were during ‘Start of firing’.

**Figure 10.**
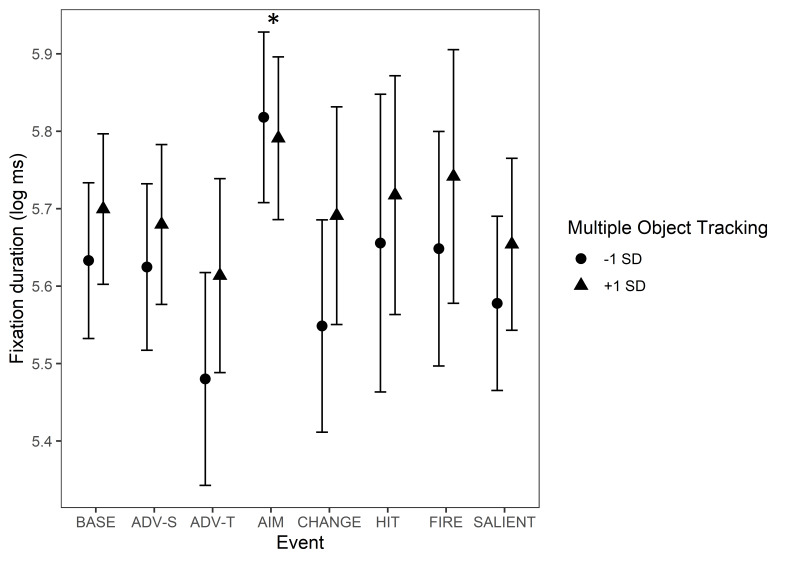
Fixation durations during game events. The circle and triangle symbols represent model estimates at values one standard deviation below and one standard deviation above the mean in MOT. The error bars denote 95% confidence intervals. Note. Significant interactions between Multiple Object Tracking and Event Type are marked with an asterisk (*). The events are abbreviated as follows: BASE = Baseline, ADV-S = Advancing (self), ADV-T = Advancing (team mate), AIM = Aiming at a target, CHANGE = Change of environment, HIT = Protagonist gets hit (bloodied visor), FIRE = Start of firing, SALIENT = Unexpected salient events.

MOT had a significant interaction with ’Aiming at a target’. In the baseline condition, participants with a higher MOT score made longer fixations than those with a lower MOT score, though not significantly so. During the ‘Aiming at a target’ event, the effect of MOT decreased and even reversed when compared to the baseline. This interaction is illustrated in Figure 10.

### Fixation Distance from Screen Center

The results of the model are reported in Table 3 in Appendix 1. There were main effects of Time, MOT and Event type. Overall, fixations were made closer to the center of the screen as time progressed. Furthermore, fixations were made closer to the center of the screen during the events of ‘Advancing (self)’, ‘Aiming at a target’, and ‘Protagonist gets hit (blood on visor)’ when compared to the baseline. Instead, during ‘Advancing (team mate)’, ‘Start of firing’, ‘Unexpected salient events’ and ‘Change of environment’, participants made fixations further away from the center than during baseline. Participants with a high score in MOT made fixations closer to the center in general.

**Figure 11.**
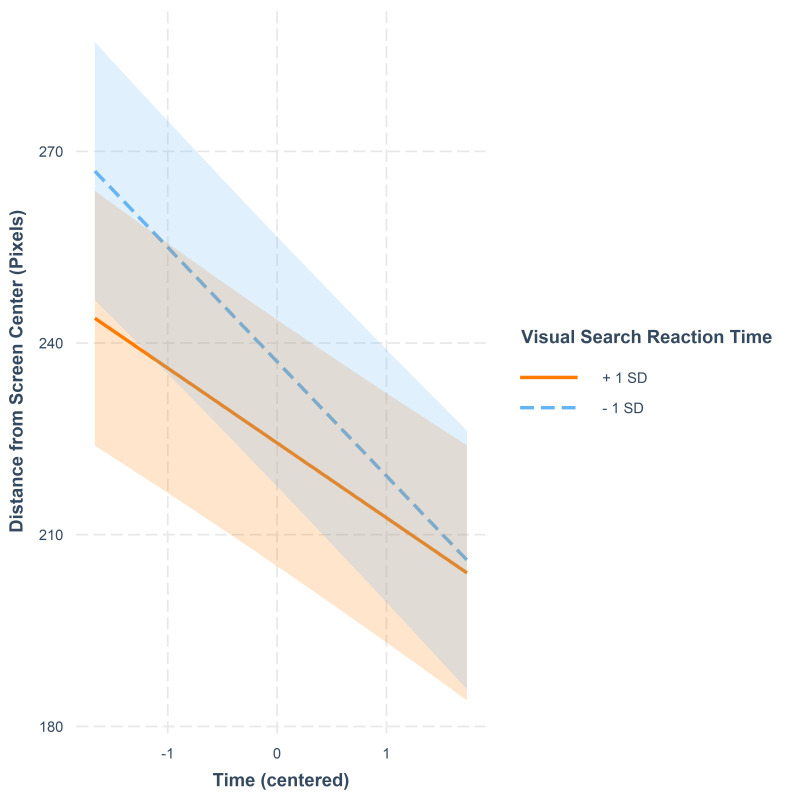
Fixation distance from screen center in pixels as a function of time. The lines represent model estimates at values one standard deviation below and one standard deviation above the mean in VS reaction time. The shaded areas denote 95% confidence intervals.

**Figure 12.**
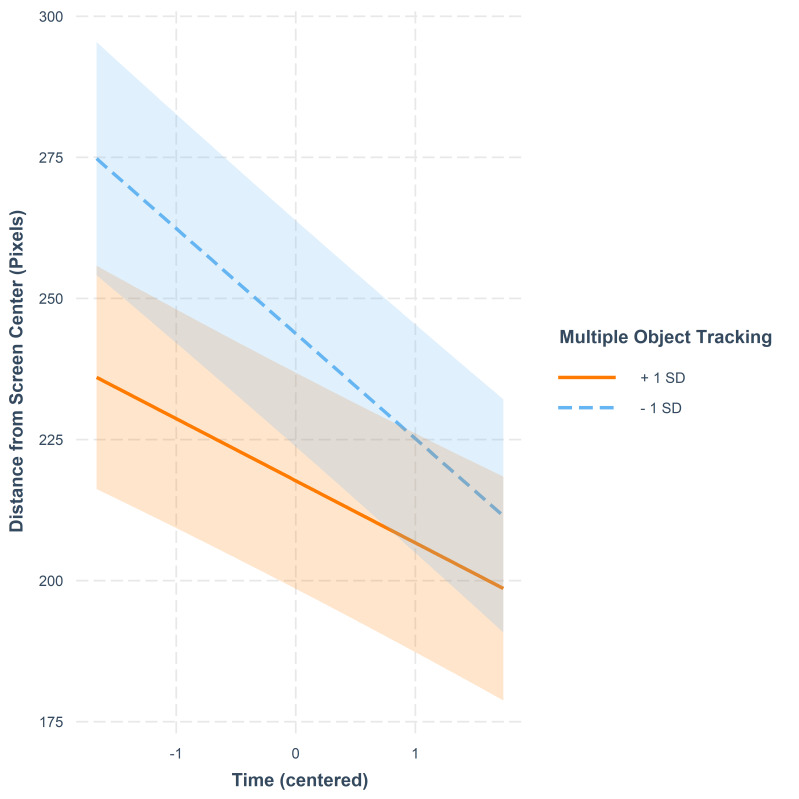
Fixation distance from screen center in pixels as a function of time. The lines represent model estimates at values one standard deviation below and one standard deviation above the mean in MOT. The shaded areas denote 95% confidence intervals.

There were VS * Time and MOT * Time interactions. These trends are illustrated in Figure 11 and Figure 12. Concerning the interaction between VS and Time, the participants who had a fast reaction time decreased their fixations’ distances more as time passed than those who had a slow reaction time. At the end of the video, their fixations fell as close to the center of the screen as of those participants’ who had a slow reaction time in VS.

A high score in MOT predicted shorter fixation distances from the center at the start of the video when compared to participants with a low score in MOT. Furthermore, those participants who had a low score in MOT showed a steeper decrease in fixation distance from the center of the screen as the video progressed. This indicates that while they started out making fixations further away from the center, they changed their strategy towards a more central watching style as time passed in a manner that was more drastic than for those participants who had a high score in MOT.

**Figure 13.**
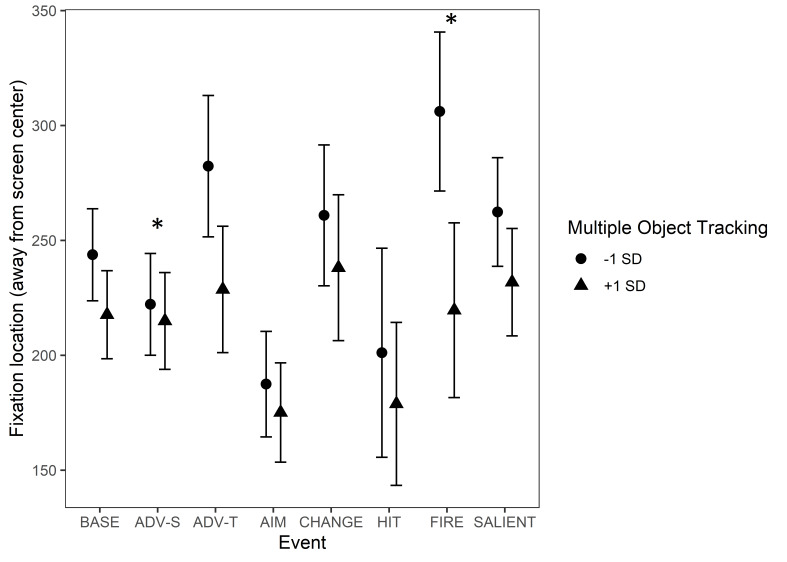
Fixation distance from screen center in pixels during game events. The circle and triangle symbols represent model estimates at values one standard deviation below and one standard deviation above the mean in MOT. The error bars denote 95% confidence intervals. Note. Significant interactions between Multiple Object Tracking and Event Type are marked with an asterisk (*). The events are abbreviated as follows: BASE = Baseline, ADV-S = Advancing (self), ADV-T = Advancing (team mate), AIM = Aiming at a target, CHANGE = Change of environment, HIT = Protagonist gets hit (bloodied visor), FIRE = Start of firing, SALIENT = Unexpected salient events.

Concerning the events, there were significant interactions between MOT and some of the events. These interactions are illustrated in Figure 13. As stated before, there was a main effect of MOT - the lower the score in MOT was, the further away from the center of the screen the fixations tended to be in during baseline. During ‘Start of firing’, this effect of MOT increased. Instead, during ‘Advancing (self)’, the effect of MOT decreased, indicating that it had less impact on how far from the screen center the fixations were.

### Saccade Amplitudes

The results of the model are reported in Table 4 in Appendix 1. There was a significant main effect of Time. The saccades got shorter as the videos progressed. There were also significant main effects for some of the events. During ‘Advancing (team mate)’ and ‘Change of environment’, the participants made longer saccades than during the baseline. Instead, during ‘Aiming at a target’ their saccades were shorter than during the baseline.

**Figure 14.**
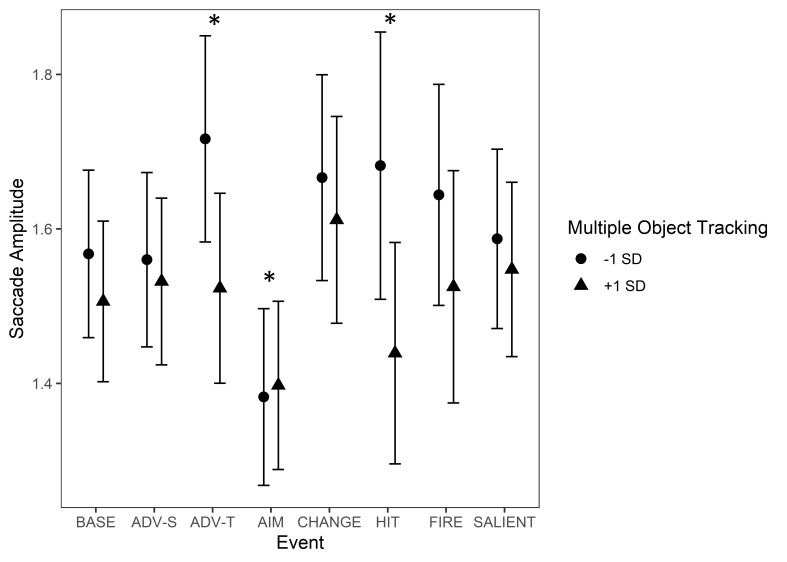
Saccade amplitudes during game events. The circle and triangle symbols represent model estimates at values one standard deviation below and one standard deviation above the mean in MOT. The error bars denote 95% confidence intervals. Note. Significant interactions between Multiple Object Tracking and Event Type are marked with an asterisk (*). The events are abbreviated as follows: BASE = Baseline, ADV-S = Advancing (self), ADV-T = Advancing (team mate), AIM = Aiming at a target, CHANGE = Change of environment, HIT = Protagonist gets hit (bloodied visor), FIRE = Start of firing, SALIENT = Unexpected salient events.

There was an interaction between MOT and some of the events. These effects are illustrated in Figure 14. In the baseline condition, longer saccades were associated with a lower MOT score. The effect of MOT increased during the events of ‘Advancing (team mate)’ and ‘Protagonist gets hit (bloodied visor)’, indicating that during these events, the weaker the skills in MOT were, the longer the saccades tended to be. Instead, during ‘Aiming at a target’, the effect of MOT decreased and even reversed.

## Discussion

In this study, we explored whether individual differences in visual attention skills are reflected in reactions to different game events during viewing of gameplay videos. We also examined how eye movement patterns develop across time, as viewers get used to the fast-paced and cluttered gameplay video. We recorded eye movements of viewers who were watching unedited gameplay videos of a First-Person Shooter (FPS) videogame. Moreover, we recorded the performance of said viewers in three visual attention tasks we considered fundamental for the viewing experience: multiple object tracking (MOT), attentional blink (AB), and visual search (VS).

The findings of this study show that individual differences in visual attention skills have an effect on dynamic scene perception. This effect presents as differing eye movements as a function of visual attention skills 1. during different events of the dynamic scene, and 2. across time, developing as time passes. Besides individual differences, we noted some typical patterns during the watching of the entire videos as well as during specific events. We will first discuss the results regarding how eye movements changed across time. Then, we will consider typical eye movement patterns during specific events of the videos. Finally, we will discuss individual differences in eye movements during viewing of gameplay videos.

### Changes in eye movements across time

One key finding of this study is that while we did find individual differences in viewing styles, the participants had an overall tendency for proceeding from what seemed to be an ambient scanning mode towards a more focal and central viewing mode during the entire length of the 6 minute dynamic scene. This was indexed as the number of fixations decreasing, saccade amplitudes shortening and fixations landing closer to the center of the screen as time progressed across the whole video. In other words, it seems that the central bias typical for dynamic scenes (16; 61) develops across time, at least during viewing of visually cluttered and relatively fast-paced gaming videos.

There are several possible reasons for this result. As the participants in this study were not particularly used to FPS games, they may have had to get accustomed to what they were seeing first by scanning the screen. After a while, they might have started to learn the spatial layout through likelihood of where different objects and people may occur (13) and whether they are relevant or not. One explanation could be simple habituation. Moreover, it is possible that the participants became more relaxed as time went on and this may have presented as a decrease in ambient scanning. In other words, the participants may have become less vigilant and less prone to checking the periphery of the screen. In any case, one should note that the results resemble the typical ambient to focal processing phases when viewing static images. It is interesting that this phenomenon seems to also present during dynamic scene perception. However, the replicability, exact mechanisms and reasons for this tendency need further studying.

### Eye Movements During Game Events

Currently, there is emerging evidence that when a new event begins in a dynamic scene, it is associated with the starting of ambient eye movements (19; 57). Our data indicates potential support for these studies, but also pinpoints that different types of events trigger distinct eye movement patterns. In our data, an ambient and less central eye movement pattern was characterized by increased numbers of fixations, decreased fixation durations, long saccades and less central fixation locations. This type of eye movement pattern was associated to some extent with the events of ‘Unexpected salient events’, ‘Start of firing’, ‘Change of environment’ and somewhat with ‘Advancing (team mate)’. However, we noted a pattern of more focal and central eye movements (indexed by decreased numbers of fixations, increased fixation durations, short saccades and fixation locations closer to the center of the screen) which showed to various extents during ‘Aiming at a target’, ‘Protagonist gets hit (bloodied visor)’ and ‘Advancing (self)’. In order to understand why different events triggered different eye movement patterns, one needs to consider the characteristics of the event types.

During the ‘Aiming at a target’ event, the number of fixations decreased, fixation durations lengthened and saccade amplitudes shortened, indicating focal eye movements. Moreover, during this event, the participants tended to concentrate their gaze on an area in the middle of the screen, which is most likely explained by the fact that there was a fixation point, namely the gun’s sight and crosshair, at that location. This finding highlights that visual aids or delegates (see 41 for a classification of different types of visual delegates of sensory phenomena used in videogames) seem to be very good at capturing attention. This seems to happen despite the fact that the participants were not playing the game themselves but merely spectated the gameplay of someone else. Besides the salient visual cue to look at the center, participants may have adopted the perspective of the player and thought that the place where the gun points to would be the most relevant (32; 36).

Another event that was highly unusual in looks was the ‘Protagonist gets hit (bloodied visor)’ event. This event, too, seemed to induce more central processing than the baseline, as indexed by lower number of fixations that tended to land closer to the center than during baseline. During this event, the screen filled with various amounts of red color (ranging from small splatters to filling the screen completely) to indicate that the protagonist was hurt. Previous studies have identified that emotional content in scenes tend to lead to more focal processing as indexed by fixations landing on a narrower area (65) and less fixations to low-level saliency cues (54), which resemble our results pertaining to this highly emotionally activating event.

Curiously, the number of fixations was lower and the fixations landed closer to the center during the event of ‘Advancing (self)’. During this event, the environment changed rapidly as the protagonist moved forwards. While it might make intuitively more sense that the eye movements would present an ambient tendency as there were many new targets to see, there are several reasons for the more central tendency. Firstly, during these events new objects appeared rapidly in the scene. Dynamic scenes tend to generate central bias more than static scenes (16; 61) as participants have a time constraint that prevents them scanning potentially irrelevant objects in the periphery. It is possible that the ‘Advancing (self)’ event simply strengthens the effect. Moreover, it is possible that theory of mind (36) comes into action or that the viewers adopt the perspective of the player (32) and look towards where the player is going, which would be approximately at the center of the screen. Along this line of thought, it is also possible that viewers are uncertain of what will happen next and look for objects that are within reach of the player, such as focusing on the foreground (13). Another very simple explanation for this phenomenon is a practical one: the viewers might want to reduce motion sickness by stabilizing the gaze when every part of the view is moving. While some of the other events also contained movement of the protagonist, the ‘Advancing (self)’ event was clearly the one in which it featured the most.

As for the events that were associated with ambient and less central eye movement patterns, there seems to be a unifying visual quality: movement in the visual field while the protagonist stays relatively still. Namely, the events that contained unexpected popping up of enemies or unexpected objects were the ones that tended to generate increased numbers of fixations, decreased fixation durations, long saccades and less central fixation locations. This result is in line with Zacks et al.’s (76) finding indicating that visual motion is often associated with events changing. Motion in general has been noted to capture attention (14; 15; 35; 42; 61; 73; 75).

The role of events on eye movements has previously been studied with naturalistic scenes of actors performing various tasks, and the scenes have been shot using a more typical camera angle such as a medium shot (19; 57). Instead, the stimuli used in this experiment consisted of a computer-generated simulation that resembled but was not equal to naturalistic dynamic scenes, and it contained visually salient cues that guided eye movements. However, the present results are in line with Pannasch (46), who showed that drastic changes in visual qualities (scene cuts and camera movements) trigger more ambient style of scanning, breaking the pattern of central tendency, which is typical during viewing of dynamic scenes. The current results are useful in pinpointing that there needs to be more research on dynamic scene event perception with different types of stimuli.

In addition to these general tendencies, there were individual differences in how viewers reacted to different types of events. Next, we will discuss how individual differences in each of the visual attention tasks were reflected in eye movements during viewing of the videos.

### Multiple Object Tracking

Individual differences in MOT performance were reflected in viewers’ eye movement patterns in several ways. During the watching of the entire 6-minute-long video, better ability to perform multiple object tracking indicated a greater tendency for central bias overall, as indicated by the main effect of MOT. However, viewers with poorer skills in MOT had a steeper decrease in fixation distance from the screen center as time passed, as indexed by the MOT x Time interaction. This result indicates that while those who were less skilled in MOT tended to inspect the screen more in its entirety at the beginning, they might have come down to the level of those who were better in MOT if the videos would have continued. However, better MOT skills were connected with a steeper decrease in number of fixations across time, again lending support to a more central tendency of viewing for those who were better at MOT. The results indicate that those who are better at MOT tend to adopt a more central style of processing overall and this tendency increases as time passes. Those who were less skilled in MOT started out as having a less central viewing style, but also moved towards more central processing as time passed, even in a more drastic progression than those who adopted the style from the beginning.

MOT skills were connected with eye movement changes in various events. As a general rule, the effect of MOT was stronger during events that contained movement or rapid change in the background, such as ‘Start of firing’. During this event, poor MOT skills were associated with the number of overall fixations being higher and the fixations landing more on the periphery as compared to those who had better MOT skills. Poorer MOT skills also showed as more fixations during ‘Change of environment’ and longer saccades during ‘Advancing (team mate)’ and ‘Protagonist gets hit (bloodied visor)’, all events that contained novel information. The results regarding MOT highlight that during events that contain movement in the background, poorer MOT skills tend to lead to a more ambient eye movement style, whereas better skills in MOT indicate a more central and focal tendency.

The results pertaining to multiple object tracking both confirm that there are individual differences in this skill (45) and that these individual differences show during viewing of a popular type of a dynamic scene, especially when a great deal of motion occurs. Previous studies on MOT have shown that tracking multiple objects is easier when targets are followed by focusing on a central location between them instead of directly on the targets (20, 21; 78). The present results suggest that this kind of strategy that characterizes good performance in MOT tasks might generalize to viewing dynamic scenes in general.

### Attentional Blink

The attentional blink task proved to be another measure that was related to individual differences in eye movement patterns observed during viewing of gameplay videos, providing further evidence that individuals differ in the susceptibility to the phenomenon (38). Less susceptibility to experiencing attentional blink was associated with the number of fixations staying more stable across time (instead of decreasing), and a decrease in fixation durations across time.

This result may be explained by considering the nature of the attentional blink itself – individuals who do not show the effect might be able to see a very rapidly presented target despite a distractor appearing before it. It makes sense that viewers who are good at this type of a task tend to pay attention to objects appearing in the periphery because they are able to see them, which may not be the case for those who are worse at perceiving them in the first place. Interestingly, people who are habitual action videogame players and thus trained with this type of a dynamic scene tend to show an attenuated attentional blink, that is, a better score in the AB task (24; 44; 80). Moreover, active videogame players tend to be better at bottom-up capture of attention in general: multiple studies have shown that active videogame players have improved target detection, i.e. they are more likely to attend to visual targets that others might miss, and also from a wider area (22; 25; 72). Even though it is hard to conclude whether videogaming improves attention or whether individuals who have the ability to resist the attentional blink are drawn towards videogaming, these results indicate that the ability to resist attentional blink is important in viewing this type of a dynamic scene. In our data, the phenomenon presents particularly well when considering how attentional blink might impact reactions to different events. For example, the participants’ score in the AB task was related to the number of fixations more than during baseline during the events of ‘Advancing (self)’, ‘Change of environment’, and ‘Start of firing’, which were all events in which there were considerable amounts of new stimuli to detect in the background.

### Visual Search

Individual differences in the VS task were reflected in changes in eye movements both across time as well as during some of the events. A faster reaction time in VS was connected with more fixations landing away from the center in the beginning of the video, and a tendency to fixate closer to the center as time went by. Moreover, participants who were fast in the VS task showed a slightly smaller decrease in the number of fixations across time. VS task performance was thus associated with switching from exploring the peripheral parts of the screen to a viewing pattern characterized by central bias.

As for reactions to different events on the video, during the ‘Start of firing’ event, the slower a participant was in VS, the shorter their fixation durations. Instead, the faster a participant was in VS, the longer the fixations were during ‘Start of firing’. The event consists of instances in which someone opens fire in the periphery, which may lead the less effective visual searchers to perform an overt search for the source, which may show as short fixations. During ‘Protagonist gets hit (bloodied visor)’ slower reaction times in VS were connected with a significant drop in the number of fixations when compared to the baseline. Instead, fast reaction times in VS were connected with the number of fixations staying stable or even increasing a little. This event presents mild to severe occlusion of the view, which may help interpret the result. Namely, it seems as though participants who had a slower reaction time in VS tended to be more fazed by the sudden onset of the blood stains on the protagonist’s visor, whereas those with a faster reaction time in VS may have continued scanning the screen to make at least some sense of what was going on.

What is interesting in these results is that even though our viewing task did not involve clear instructions to perform a search, performance in the VS task was associated with individual differences in the viewing patterns. The VS score thus reflects something that is more general than performing a search for a specific target in an array. The present findings indicate that the VS task taps into the ability to adapt to the visual environment and be able to control one’s attentional resources in cluttered dynamic visual settings (e.g., 9).

### Limitations

There are some limitations to the results found in this study. As many of the events of the videos contained sound effects, such as gunshots, explosions, yelling, and spoken instructions from the team leader, it is possible that the differences in eye movements we noted between events could have been affected by auditory cues. Moreover, we did not control the participants’ English language skills (they were non-native speakers of English), but considered videogame watching as a purely visual task even though the audio played in the background. One might make the argument that sounds or speech are enough to affect eye movements and may have conflicted our results. While this should be studied further in future explorations of this topic, we would like to note that the sounds appeared systematically in connection with the events. For example, during the ‘Start of firing’ event, there were always sudden gunshots that did not appear as suddenly during other events.

Another limitation is that even though the events on the video were initially categorized as meaningful actions (19), we did not analyze the narrative structure of the video in more detail. Smith (60) has pointed out that one endogenous factor that may direct attention during film watching is narrative, and future endeavors should focus more on the effect of narrative comprehension on visual attention. As the material utilized here contained only a very thinly thread narrative, we did not consider it at this time. This should be explored more in further studies on videogaming videos.

Moreover, one should note that the stimuli used in this study consisted of highly emotional content. While it is beyond the scope of this study to further explore the effects of emotional responses on eye movements in dynamic scene viewing, this could also be a potential area for further study, as emotional content has been known to capture attention and lead to focal processing (54; 65). Because of the emotional content, it is also somewhat difficult to compare this study to other studies that have utilized everyday scenarios and actions, as the actions of the videos in this study consisted of highly unlikely content, such as shooting at people.

In this study, we were interested in how individual differences in visual attention skills affect the perception of a cluttered dynamic scene. As the study may also be of interest to readers interested in the training effects of videogames, we would like to note that we did not test active players at this time. This was because we wanted to avoid rehearsal effects with the particular stimulus we used – in short, we wanted to make sure that the participants were not accustomed to the stimulus, which might have affected the results. In future studies, it might be fruitful to also study expert videogame players’ eye movements. This would help in determining for example the relevant targets that viewers make fixations to, or typical eye movement patterns for a certain type of event. Methodology such as used by Wang et al. (79) to generate points of interest could be fruitful for this purpose. One could compare how well novice players of differing visual attention skills are able to find relevant points of interests, and whether the time course of these events (hectic or slow gameplay) affects differences in eye movements. These types of studies would provide a more solid theory base for future study endeavors, as well as be of practical use in for example e-sports training programs.

Regarding short-term rehearsal effects, in future studies it might be worthwhile to also control the order effects of either playing or watching a game, or to build the study so that it would only include watching of the game.

Finally, we acknowledge that this study is somewhat exploratory in nature, and the analyses and results presented may seem complex. However, it is our hope that the current descriptions may aid further endeavors in studying dynamic scene perception or videogames. An interesting venture for studying the role of individual differences in dynamic scene perception would be to look at location-based attentional synchrony or clustering of gaze data (as done by for example 61).

### Conclusions

Previous studies on the effect of events on eye movements (19; 57) have examined how event boundaries are segmented and perceived, whereas the present study studied eye movements as a reaction to different types of events in a videogame setting. Our approach is similar to other studies that have annotated FPS game events (34; 37; 43; 50; 71) and our events matched them well. The present results extend these lines of research by demonstrating that different events may trigger different eye movement patterns during dynamic scene viewing, and that there are individual differences in these reactions.

The findings regarding individual differences are in line with and extend the results of other studies about individual differences in scene perception as indexed by eye movements (2; 12; 27; 51; 56). However, to our knowledge, individual differences in the specific visual attention tasks studied here have not been explored before in connection with eye movements during dynamic scene perception. Previous studies have examined attentional synchrony between individuals when watching edited (42; 58) and unedited (16) videos.

Theoretical views on individual differences in eye movements assume that there is a global component, such as general intelligence, that is associated with individual differences in eye movements during scene perception (27). Our results offer complimentary support but also somewhat contradict Hayes’ and Henderson’s (27) finding that cognitive skills are connected with a central viewing style. In our data, MOT performance was indeed connected with a more central viewing style overall. However, resistance to the AB effect seemed to be associated with a less central viewing style, especially during particular events. The results may be explained by the fact that we used tasks that were specific to visual attention, whereas Hayes and Henderson (17) used more general skill tasks. Also, Hayes and Henderson (17) did not find any results on global metrics such as fixation duration or frequency and instead found them on scan patterns. Our results highlight both that more research on individual differences is needed, and that future research should focus on different aspects of visual attention.

Our focus on gameplay videos proved to be fruitful in revealing how different individuals deal with the perceptual demands of viewing dynamic scenes. Tatler et al. (66) have pointed out that as scene complexity increases, it is more likely that top-down processes are engaged. It seems clear that the visual qualities of the FPS gameplay video are straining enough to bring about the role of individual differences in the ability to control attention in a top-down manner.

The finding that there are individual differences in the ability to control visual attention that affect spectating of gameplay videos may to some extent explain differences in enjoyment of videogame streaming or eSports spectatorship. Perhaps some individuals are better able to follow games because of their visual attention skills that guide their attention towards relevant aspects of the game.

### Ethics and Conflict of Interest

This study has been evaluated by the Ethics Committee for Human Sciences at the University of Turku. According to the review statement, it has been considered to pose no major harm for participants.

The authors declare no conflicts of interest.

### Acknowledgements

Funding: This work was supported in part by Finnish Cultural Foundation (Varsinais-Suomi Regional Fund), Kone Foundation, TOP foundation, The Mannerheim League for Child Welfare Foundation, HPY Research Foundation, and Finnish Foundation for Psychiatric Research.

We would like to thank Dr. Lauri Oksama for scripting the MOT task.

## Appendix 1: Model Tables

**Table 1.** Model for Number of Fixations.

Random Effects	n	Variance	SD	
Participant (Intercept)	38	.02	.14	
Game Mission (Intercept)	4	. 0003	. 02	
				
Fixed Effects	Estimates	95% CI	*z*	*p*
(Intercept)	4.85	4.80 – 4.89	198.37	**<.001**
Time	-.03	-.03 – -.03	-59.93	**<.001**
Advancing (self)	-.03	-.03 – -.03	-16.58	**<.001**
Advancing (team mate)	-.02	-.03 – -.01	-5.09	**<.001**
Aiming at a target	-.04	-.04 – -.03	-18.19	**<.001**
Change of environment	.01	-.00 – .01	1.22	.223
Protagonist gets hit (bloodied visor)	-.03	-.04 – -.01	-4.05	**<.001**
Start of firing	.08	.07 – .09	16.57	**<.001**
Unexpected salient events	.01	.01 – .02	5.64	**<.001**
MOT	-.03	-.08 – .02	-1.18	.238
VS	-.03	-.08 – .02	-1.25	.211
AB	.02	-.03 – .07	.86	.388
Time * MOT	-.00	-.00 – -.00	-6.64	**<.001**
Time * VS	-.00	-.00 – -.00	-2.24	**.025**
Time * AB	.02	.02 – .02	36.18	**<.001**
Advancing (self) * MOT	.01	.01 – .01	5.09	**<.001**
Advancing (team mate) * MOT	.01	.01 – .02	3.74	**<.001**
Aiming at a target * MOT	.02	.01 – .02	7.55	**<.001**
Change of environment * MOT	-.03	-.04 – -.02	-7.38	**<.001**
Protagonist gets hit (bloodied visor) * MOT	.01	-.00 – .03	1.89	.059
Start of firing * MOT	-.03	-.04 – -.02	-5.69	**<.001**
Unexpected salient events * MOT	-.00	-.01 – .00	-.71	.477
Advancing (self) * VS	.00	-.00 – .00	.25	.806
Advancing (team mate) * VS	.00	-.01 – .01	.14	.887
Aiming at a target * VS	-.00	-.01 – .00	-1.01	.314
Change of environment * VS	-.01	-.02 – .00	-1.69	.092
Protagonist gets hit (bloodied visor) * VS	-.05	-.07 – -.03	-5.00	**<.001**
Start of firing * VS	.01	-.00 – .02	1.75	.081
Unexpected salient events * VS	-.00	-.01 – .00	-.91	.360
Advancing (self) * AB	.01	.00 – .01	3.31	**.001**
Advancing (team mate) * AB	-.02	-.02 – -.01	-4.16	**<.001**
Aiming at a target * AB	-.01	-.01 – -.00	-3.14	**.002**
Change of environment * AB	.02	.01 – .02	3.52	**<.001**
Protagonist gets hit (bloodied visor) * AB	-.03	-.04 – -.02	-4.65	**<.001**
Start of firing * AB	.01	.00 – .02	2.88	**.004**
Unexpected salient events * AB	-.00	-.01 – .00	-1.10	.270

**Table 2.** Model for Fixation Duration.

Random Effects	n	Variance	SD	
Participant (Intercept)	38	.04	.20	
Game Mission (Intercept)	4	.001	.04	
Residual		.51	.71	
				
Fixed Effects	Estimates	95% CI	*t*	
(Intercept)	5.67	5.59 – 5.74	**151.18**	
Time	-.01	-.02 – .00	-1.84	
Advancing (self)	-.01	-.04 – .02	-.94	
Advancing (team mate)	-.12	-.18 – -.06	**-3.70**	
Aiming at a target	.14	.11 – .17	**8.26**	
Change of environment	-.05	-.12 – .02	-1.31	
Protagonist gets hit (bloodied visor)	.02	-.08 – .12	.40	
Start of firing	.03	-.06 – .11	.67	
Unexpected salient events	-.05	-.09 – -.01	**-2.57**	
VS	.03	-.04 – .10	.84	
MOT	.03	-.03 – .10	.99	
AB	-.03	-.10 – .04	-.92	
Time * VS	-.00	-.01 – .01	-.62	
Time * MOT	-.00	-.01 – .01	-.18	
Time * AB	-.01	-.02 – -.00	**-2.24**	
Advancing (self) * VS	-.03	-.06 – .00	-1.71	
Advancing (team mate) * VS	.03	-.04 – .11	.80	
Aiming at a target * VS	.02	-.02 – .06	1.16	
Change of environment * VS	-.02	-.09 – .05	-.54	
Protagonist gets hit (bloodied visor) * VS	.03	-0.11 – 0.17	.39	
Start of firing * VS	-.10	-.20 – -.01	**-2.11**	
Unexpected salient events * VS	-.02	-.06 – .01	-1.25	
Advancing (self) * MOT	-.01	-.03 – .02	-.39	
Advancing (team mate) * MOT	.03	-.03 – .10	1.05	
Aiming at a target * MOT	-.05	-.08 – -.01	**-2.75**	
Change of environment * MOT	.04	-.03 – .11	1.05	
Protagonist gets hit (bloodied visor) * MOT	-.00	-.11 – .10	-.04	
Start of firing * MOT	.01	-.08 – .11	.28	
Unexpected salient events * MOT	.00	-.04 – .05	.24	
Advancing (self) * AB	.00	-.03 – .03	.07	
Advancing (team mate) * AB	.04	-.02 – .11	1.24	
Aiming at a target * AB	.02	-.01 – .06	1.29	
Change of environment * AB	-.02	-.09 – .05	-.64	
Protagonist gets hit (bloodied visor) * AB	.10	-.00 – .20	1.91	
Start of firing * AB	.01	-.08 – .09	.16	
Unexpected salient events * AB	-.00	-.04 – .04	-.15	

*Note. t-*values > 1.96 are in boldface to indicate statistical significance.

**Table 3.** Model for Fixation Distance from Screen Center.

Random Effects	n	Variance	SD
Participant (Intercept)	38	816.80	28.58
Game Mission (Intercept)	4	189.00	13.75
Residual		31567.50	177.67
			
Fixed Effects	Estimates	95% CI	*t*
(Intercept)	230.73	214.22 – 247.24	**27.39**
Time	-14.80	-16.88 – -12.73	**-13.99**
Advancing (self)	-12.15	-19.46 – -4.83	**-3.26**
Advancing (team mate)	24.78	9.03 – 40.53	**3.08**
Aiming at a target	-49.43	-57.61 – -41.26	**-11.85**
Change of environment	18.79	1.49 – 36.09	**2.13**
Protagonist gets hit (bloodied visor)	-40.72	-65.36 – -16.07	**-3.24**
Start of firing	32.13	11.22 – 53.04	**3.01**
Unexpected salient events	16.37	6.79 – 25.95	**3.35**
MOT	-13.04	-23.59 – -2.48	**-2.42**
VS	-6.37	-16.60 – 3.86	-1.22
AB	-5.23	-15.31 – 4.85	-1.02
Time * MOT	3.81	1.68 – 5.93	**3.51**
Time * VS	3.09	.90 – 5.28	**2.76**
Time * AB	1.72	-.47 – 3.91	1.54
Advancing (self) * MOT	9.42	2.17 – 16.67	**2.55**
Advancing (team mate) * MOT	-13.78	-29.32 – 1.76	-1.74
Aiming at a target * MOT	6.86	-1.45 – 15.18	1.62
Change of environment * MOT	1.65	-16.06 – 19.36	.18
Protagonist gets hit (bloodied visor) * MOT	1.92	-24.44 – 28.29	.14
Start of firing * MOT	-30.20	-53.09 – -7.31	**-2.59**
Unexpected salient events * MOT	-2.23	-12.23 – 7.78	-.44
Advancing (self) * VS	.12	-7.70 – 7.94	.03
Advancing (team mate) * VS	-8.85	-27.53 – 9.83	-.93
Aiming at a target * VS	-3.08	-12.50 – 6.33	-.64
Change of environment * VS	-4.12	-22.35 – 14.11	-.44
Protagonist gets hit (bloodied visor) * VS	-3.18	-38.74 – 32.37	-.18
Start of firing * VS	17.87	-6.11 – 41.86	1.46
Unexpected salient events * VS	3.12	-6.22 – 12.46	.65
Advancing (self) * AB	-4.23	-11.71 – 3.25	-1.11
Advancing (team mate) * AB	-4.23	-20.30 – 11.84	-.52
Aiming at a target * AB	-5.79	-14.77 – 3.18	-1.26
Change of environment * AB	-.11	-17.66 – 17.44	-.01
Protagonist gets hit (bloodied visor) * AB	-22.55	-47.95 – 2.86	-1.74
Start of firing * AB	12.10	-9.19 – 33.39	1.11
Unexpected salient events * AB	4.25	-5.38 – 13.89	.87

*Note. t-*values > 1.96 are in boldface to indicate statistical significance.

**Table 4.** Model for Saccade Amplitude.

Random Effects	n	Variance	SD
Participant (Intercept)	38	.02	.14
Game Mission (Intercept)	4	.007	.08
Residual		.35	.59
			
Fixed Effects	Estimates	95% CI	*t*
(Intercept)	1.54	1.44 – 1.63	**32.41**
Time	-.02	-.03 – -.01	**-5.64**
Advancing (self)	.01	-.02 – .03	.74
Advancing (team mate)	.08	.03 – .14	**3.10**
Aiming at a target	-.15	-.17 – -.12	**-10.78**
Change of environment	.10	.04 – .16	**3.47**
Protagonist gets hit (bloodied visor)	.02	-.06 – .11	.57
Start of firing	.05	-.02 – .12	1.35
Unexpected salient events	.03	-.00 – .06	1.89
VS	-.02	-.07 – .03	-.78
MOT	-.03	-.08 – .02	-1.17
AB	.02	-.03 – .07	.81
Time * VS	-.00	-.01 – .01	-.22
Time * MOT	.00	-.00 – .01	.57
Time * AB	.01	-.00 – .01	1.86
Advancing (self) * VS	.01	-.02 – .04	.74
Advancing (team mate) * VS	.03	-.03 – .09	.89
Aiming at a target * VS	.01	-.02 – .04	.74
Change of environment * VS	-.02	-.08 – .04	-.60
Protagonist gets hit (bloodied visor) * VS	.08	-.04 – .20	1.32
Start of firing * VS	-.01	-.09 – .07	-.20
Unexpected salient events * VS	-.01	-.04 – .02	-.61
Advancing (self) * MOT	.02	-.01 – .04	1.36
Advancing (team mate) * MOT	-.07	-.12 – -.01	**-2.49**
Aiming at a target * MOT	.04	.01 – .07	**2.76**
Change of environment * MOT	.00	-.06 – .06	.11
Protagonist gets hit (bloodied visor) * MOT	-.09	-.18 – -.00	**-2.04**
Start of firing * MOT	-.03	-.10 – .05	-.74
Unexpected salient events * MOT	.01	-.02 – .04	.65
Advancing (self) * AB	-.01	-.03 – .02	-.49
Advancing (team mate) * AB	.01	-.05 – .06	.31
Aiming at a target * AB	-.01	-.04 – .02	-.71
Change of environment * AB	-.04	-.10 – .01	-1.50
Protagonist gets hit (bloodied visor) * AB	.01	-.07 – .09	.25
Start of firing * AB	-.02	-.09 – .05	-.63
Unexpected salient events * AB	.01	-.02 – .04	.68

*Note. t-*values > 1.96 are in boldface to indicate statistical significance..
